# 2087. Prescription Adherence and Persistence on Oral Pre-exposure Prophylaxis (PrEP) Among PrEP-Naive (PN) Individuals After F/TAF Approval in the United States (US)

**DOI:** 10.1093/ofid/ofac492.1709

**Published:** 2022-12-15

**Authors:** Rick A Elion, Joshua Gruber, Janna Radtchenko, Megan Dunbar, Kenneth H Mayer, Gregory Huhn, Karam Mounzer, Anthony Mills

**Affiliations:** Trio Health, Louisville, Colorado; Gilead Sciences, Forest City, California; Trio Health, Louisville, Colorado; Gilead Sciences, Forest City, California; Fenway Health, Boston, Massachusetts; The Ruth M. Rothstein CORE CENTER, Chicago, Illinois; Philadelphia Fight Community Health Centers, Philadelphia, Pennsylvania; Men's Health, Los Angeles, California

## Abstract

**Background:**

We evaluated utilization of emtricitabine/tenofovir disoproxil fumarate or tenofovir alafenamide (F/TDF, F/TAF) among PN after the approval of F/TAF for PrEP in the US.

**Methods:**

EMR and dispensing data from Trio Health HIV Research Network were used for this retrospective observational study. The study included HIV-negative PN ≥ 18 years with first dispense of daily oral PrEP (≥30-day supply) between 10/19-5/21 followed for ≥6 mo; individuals with hepatitis B or post-exposure prophylaxis were excluded. Prescription adherence, measured as proportion of days covered (PDC; mean and proportion with PDC ≥50, 70, and 80%) and time to regimen discontinuation (no drug >3 mo) or switch (TRD; Kaplan-Meier analysis) were compared between regimens. Characteristics associated with PDC and time to first regimen stop (switch/discontinuation) were evaluated using generalized linear regression and Cox proportional hazard models, respectively.

**Results:**

Of 1330 PrEP starts, 86% (1144) were dispensed F/TAF vs 14% F/TDF (186). Baseline characteristics differed by regimen [Table 1]. While PDC was similar for both regimens, F/TAF had higher number of dispenses and mean days supplied vs F/TDF; mean days of follow-up were similar [Table 1]. F/TAF users had longer TRD (mean 20.2 vs 8.5 mo, Log-rank p< .001); median TRD was 3.9 mo for F/TDF and not reached for F/TAF [Figure 1]. A higher proportion of PN on F/TDF discontinued (46% vs 24% F/TAF) and switched (26% vs 2% F/TAF) their regimen (both p< .001).

After accounting for gender, race, payer, age, high-risk behavior, F/TDF had a higher risk of discontinuation or switch (HR=4.9 CI 3.9-6.2); Black race was also associated with higher risk of discontinuation or switch [Table 2]. Results were similar when considering only discontinuation (censoring at time of switch or loss to follow up). Older age was identified as the primary driver of PDC controlling for other factors [Table 2].

**Table 1.**
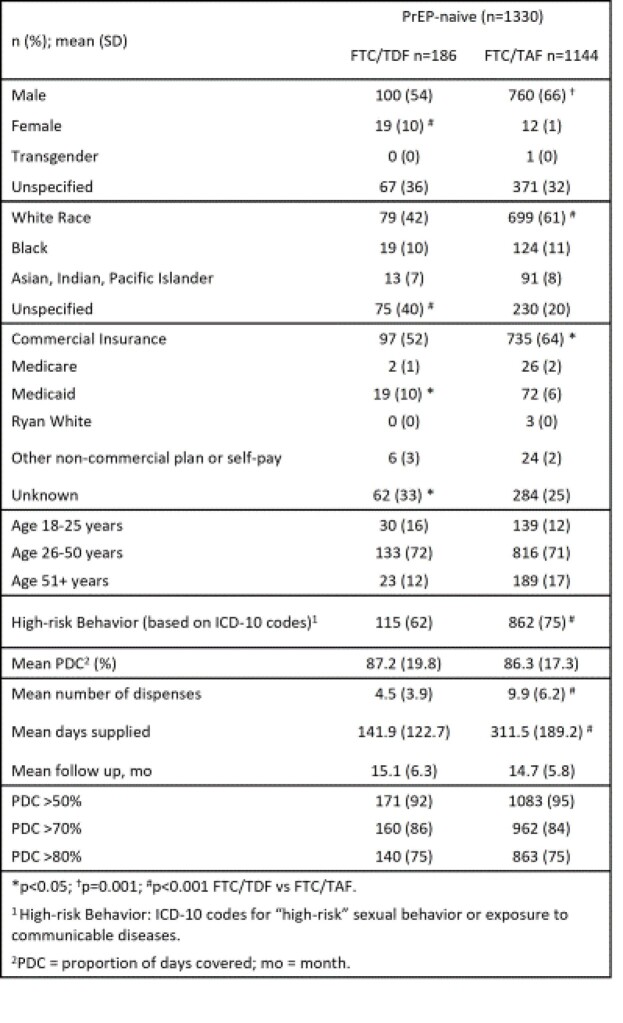
Characteristics of PN Individuals Dispensed Oral PrEP After October 2019 and PDC on First PrEP Regimen

**Figure 1.**
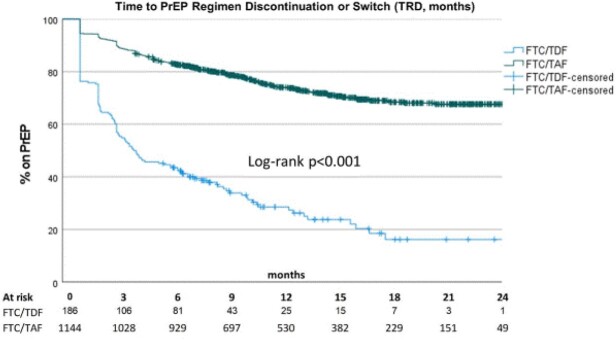
Time to PrEP Regimen Discontinuation or Switch (TRD, months)

**Table 2.**
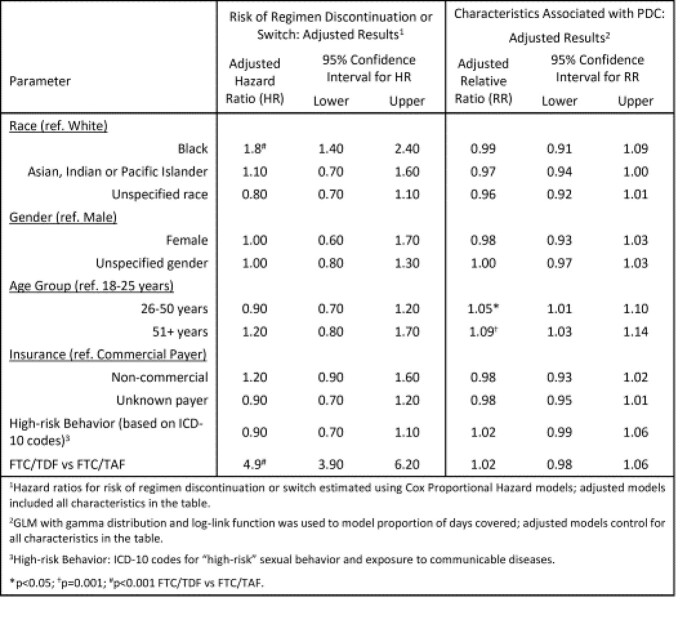
Risk of First Oral PrEP Regimen Discontinuation or Switch and Characteristics Associated with Higher PDC

**Conclusion:**

In this study PN adults dispensed F/TAF had greater number of dispenses, mean days supplied, and were less likely to discontinue or switch from F/TAF compared to F/TDF. Older age was the primary driver of increased PDC when considering other factors, including demographics, insurance and regimen.

**Disclosures:**

**Rick A. Elion, MD**, Gilead Sciences: Advisor/Consultant|Trio Health: Employee|ViiV: Advisor/Consultant **Joshua Gruber, PhD**, Gilead Sciences: Employee **Janna Radtchenko, MBA**, Trio Health: Employee **Megan Dunbar, PhD**, Gilead Sciences: Employee **Kenneth H. Mayer, MD**, Gilead: Advisor/Consultant|Merck: Advisor/Consultant|ViiV: Advisor/Consultant **Gregory Huhn, MD, MPHTM**, Eli Lilly: Advisor/Consultant|Eli Lilly: Grant/Research Support|Gilead: Advisor/Consultant|Gilead: Grant/Research Support|Jannsen: Advisor/Consultant|Jannsen: Grant/Research Support|Merck: Advisor/Consultant|Viiv: Advisor/Consultant|Viiv: Grant/Research Support **Karam Mounzer, MD**, Epividian: Advisor/Consultant|Gilead Sciences: Advisor/Consultant|Gilead Sciences: Grant/Research Support|Janssen: Advisor/Consultant|Janssen: Grant/Research Support|Merck: Advisor/Consultant|Merck: Grant/Research Support|Trio Health: Advisor/Consultant|ViiV: Advisor/Consultant|ViiV: Grant/Research Support **Anthony Mills, MD**, Gilead Sciences: Advisor/Consultant|Gilead Sciences: Grant/Research Support|Merck: Advisor/Consultant|Merck: Grant/Research Support|ViiV: Advisor/Consultant|ViiV: Grant/Research Support.

